# Smart transparent surfaces for energy-efficient buildings: enabling 5G mmWave connectivity with multispectral compatibility

**DOI:** 10.1038/s41598-026-50027-x

**Published:** 2026-04-22

**Authors:** Dat Tien Nguyen, Ji Hun Shin, Hyeon-June Kim, Chang Won Jung

**Affiliations:** Department of Semiconductor Engineering, Seoul National University of Science and Technology, Seoul, 01811 South Korea

**Keywords:** Engineering, Materials science, Optics and photonics

## Abstract

This paper presents a glass-penetrating transparent surface (GPTS) that provides multispectral compatibility across visible light (VIS), infrared (IR), and ultraviolet (UV) bands, while improving transmission in the 5G millimeter-wave (mmWave) band. For building applications, these techniques are critical to efficiently deploy on-premises mmWave fixed wireless access (FWA) as well as wireless communications with outdoor-to-indoor and indoor-to-outdoor connectivity. To optimize transmission and angular stability, various frequency-selective surface (FSS) designs based on a metallic layer (ML) on glass (GPTS-ML) are first investigated. The ML was then replaced with two types of advanced transparent coatings PEI/Ag/PEDOT: PSS: a transparent electrode (TE) and a low-E (LE) coating with sheet resistances of 9.2 Ω/sq and 5 Ω/sq, respectively. Both structures achieve a transmission loss below 4.7 dB at 28 GHz, while maintaining high VIS transmittance ($\:{T}_{vis}$) and simultaneously reducing IR transmittance ($\:{T}_{IR}$) and UV transmittance ($\:{T}_{UV}$). GPTS-TE provides $\:{T}_{vis}$ of approximately 66% with balanced $\:{T}_{UV}$ of 40% and $\:{T}_{IR}$ of 44%, whereas GPTS-LE exhibits stronger IR/UV blocking, with $\:{T}_{IR}$ of 19% and $\:{T}_{UV}$ of 29.5%, while sustaining low-loss mm-wave transmission. These results illustrate a path towards multi-spectral surface applications that simultaneously support energy efficiency, user comfort, and next generation 5G mmWave FWA services. Our work highlights the potential of transparent metasurfaces as a foundational element in smart buildings and urban communications infrastructure, thereby enabling sustainable and connected living environments.

## Introduction

Multispectral-compatibility technologies, including radar–infrared, infrared–visible, and radar–infrared–visible stealth, have been applied in the military scale^1,2^. Among them, the infrared-visible application—also referred to as low-emissivity (low-E) windows—has been extensively used for external glazing in both residential and commercial buildings. This approach is considered one of the most effective ways to reduce energy loss through windows in modern buildings while maintaining visible light. Most exterior low-E windows in modern buildings use double-pane glass with a low-E coating on the interior surface and insulating air gaps between the glass panes^3^. This low-E coating acts as an electromagnetic (EM) shield and was a primary cause of signal attenuation or transmission loss ($\:{S}_{21}$) in indoor fixed wireless access (FWA) systems^4,5^.

FWA enables network operators to deliver ultra-high-speed 5G mmWave broadband in areas where fixed-line deployment is difficult or expensive^6^. 5G mmWave FWA can provide fiber-like capacity, and beamformed signals from the access unit (AU) can serve customer premises equipment (CPE). CPE refers to devices installed at the user premises, such as routers (modems), that receive wireless signal from the.

**Fig. 1:**
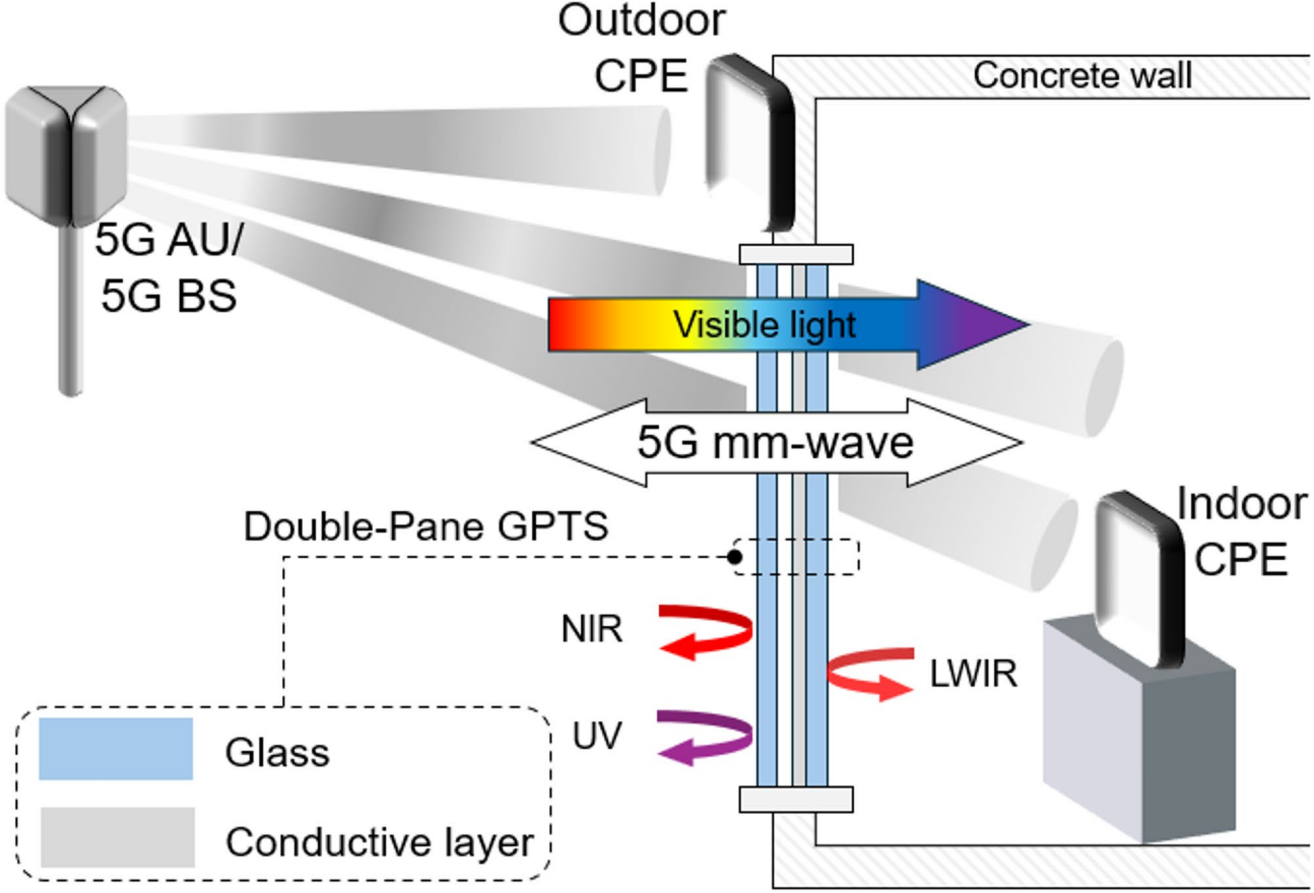
Concept of enhanced 5G mmWave FWA transmission and wireless communications for residential and commercial buildings with multi-spectrum compatibility. The proposed double-pane glass-penetrating transparent surface (GPTS) blocks ultraviolet (UV, 100–380 nm), near-infrared (NIR, 780–3000 nm), and long-wave infrared (LWIR, 2.5–50 μm) while transmitting visible light (380–780 nm) and 5G mmWave.

AU. An outdoor CPE places its antenna outside the building to minimize signal attenuation. On the other hand, indoor CPE can switch flexibly between ethernet and cellular connections, which improves robustness to external conditions [see Fig. 1]. However, indoor deployment results in higher $\:{S}_{21}$ because low-E window coatings significantly attenuate the signal^7^.

To mitigate this issue, several studies have investigated improvements in signal transmission by employing aperture-type frequency selective surfaces (FSSs)^8^, where the coating removal area (CRA) is minimized (< 5%) to preserve the multispectral compatibility of low-E coating^9–16^^[,17^. For FSS designs, these metasurfaces typically employ patch^7,14,15^, ring^16^, or hexagonal^17^ structures while overlooking the analysis of the quality factor (Q-factor), a crucial parameter for transmission efficiency and angular stability. Another approach involves employing antireflection coatings^7,18^ or installing signal repeaters^19^.

This study proposes a double-pane GPTS combined with a low-E coating to provide multispectral compatibility by simultaneously blocking IR–UV while transmitting visible light and 5G mmWave (n257, 26.5–29.5 GHz). For the selection of the FSS structure, several representative FSS configurations based on ML were to investigate the impact of structural elements on performance stability. A modified structure, referred to as GPTS-ML, has been proposed based on a cross-dipole design. Both simulation and measurement results indicate that the proposed GPTS-ML exhibits high transmission and angular stability. Subsequently, the ML was first replaced with the TE, forming GPTS-TE, and then with the LE coating, forming GPTS-LE, to align with the objectives of the proposed work. The key novelties of this work are as follows:

1. Enhance transmission and angular stability: Simulation designs of various FSS structures for low-E glass applications were first carried out to evaluate the proposed design. Both simulation and measurement results indicate that the proposed GPTS-ML exhibits better transmission and angular stability compared to other FSS structures reported in^8,9^^13,14^^[,15^.
2. Coating material selection: This work is the first to introduce the PEI/Ag/PEDOT: PSS (PAP) coating for FSS-based designs, aiming to enhance EM wave transmission through low-E glass^20^. The PAP coating exhibits superior optical and physical properties, featuring surface-level density at the atomic scale. Thus, PAP exhibits outstanding performance, including a bending radius below 1 mm, $\:{T}_{vis}$ greater than 95%, and a sheet resistance ($\:{R}_{s}$) lower than 10 Ω/sq.

## Double-pane GPTS modelling

### Selection of single-pane GPTS element

**Fig. 2:**
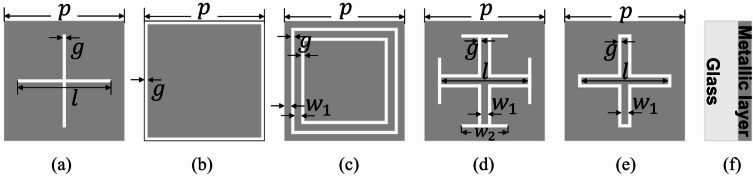
Optimized structure of single-pane GPTSs. (**a**) Case (1) (**b**) Case (2) (**c**) Case (3) (**d**) Case (4) (**e**) Case (5) (**f**) Side view.

**Table 1:** Dimensions and coating area removed for single-pane GPTS from case 1 to case 5.

Type	FSS Parameters					CRA (%)
	$\:\boldsymbol{p}$ ($\:\boldsymbol{m}\boldsymbol{m}$)	$\:\boldsymbol{g}$ ($\:\boldsymbol{m}\boldsymbol{m}$)	$\:\boldsymbol{l}$ ($\:\boldsymbol{m}\boldsymbol{m}$)	$\:{\boldsymbol{w}}_{1}$ ($\:\boldsymbol{m}\boldsymbol{m}$)	$\:{\boldsymbol{w}}_{2}$ ($\:\boldsymbol{m}\boldsymbol{m}$)	
Case 1^9^	3.35	0.03	3.3	–	–	1.75
Case 2^14^–^15^	1.25	0.03	–	–	–	9.36
Case 3^13^	1.75	0.03	–	0.1	–	10.9
Case 4^8^	2.3	0.03	1.64	0.35	1	5.1
Case 5 [This work]	3	0.03	1.94	0.35	–	2.6

The FSS provides a solution for improving wave transmission and angular stability. This requires careful consideration in the design process. Since the FSS has to be aperture-type structure, it should be designed in a way that minimizes the CRA on the glass surface to reduce the degradation of the optical properties of low-E glass. Due to the presence of the coating layer, achieving a strongly resonant FSS in this case is not feasible, as it also depends on the $\:{R}_{s}$ and the CRA^4^. However, the transmission capability can be improved based on the equivalent circuit model by considering the ML design^5^. Therefore, before proceeding with the proposed FSS design, several simulations were conducted to compare the Q-factor of FSS structures based on the ML^8,9^^13,14^^[,15^.

In Fig. 2a, b, c and d respectively illustrate the conventional cross dipole (case 1), the square patch (case 2), the double square loop (case 3), the Jerusalem cross dipole (case 4), and the proposed modified cross dipole (case 5), while Fig. 2f shows the side view of the five cases, with their dimensions and the CRA detailed in Table 1. The ML has a thickness of 35 μm and is placed on a 3.3 mm-thick glass substrate, which has a dielectric constant ($\:{\epsilon\:}_{r}$) of 4.6 and a loss tangent (tan) of 0.0037.

**Fig. 3:**
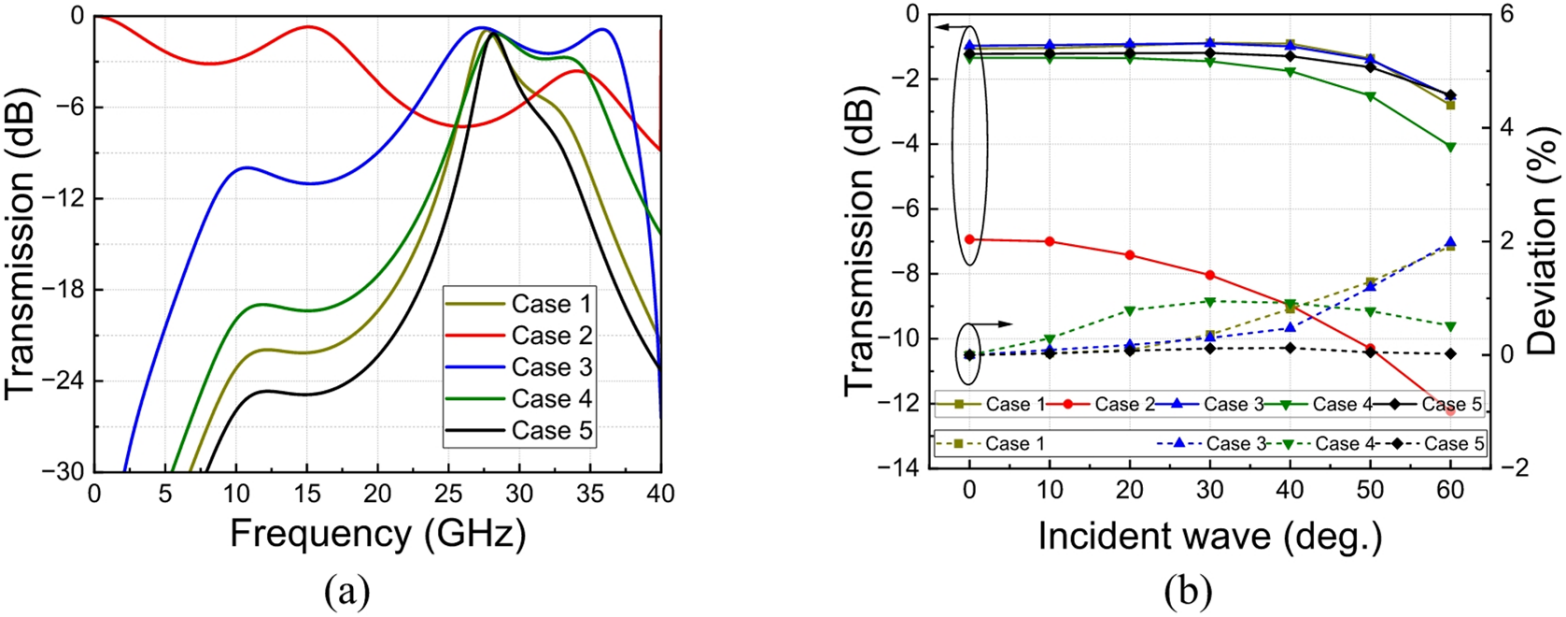
Simulation results of $\:{S}_{21}$ for the structures shown in Fig. 2. (**a**) Frequency response from DC to 40 GHz. (**b**) Deviation in $\:{\phi ^{TE}}$ mode at 28 GHz.

The simulation results for the cases in Fig. 2 are analyzed in terms of transmission coefficients, peak transmission at 28 GHz, and deviation with transverse electric ($\:{\phi\:}^{TE})$ mode, as shown in Fig. 3. The gap between the metallic lines ($\:g$) is fixed at 30 μm, while the unit cell size ($\:p$) varies depending on the electric field distribution of each structure to achieve resonance at 28 GHz. In the full-wave simulations, the ML was replaced by copper and analyzed using the frequency-domain solver with a unit-cell model in CST Studio Suite. First, when considering transmission enhancement, cases 1, 3, 4, and 5 exhibit a bandpass response at n257 band with low $\:{S}_{21}$ of approximately 1 dB at 28 GHz, whereas case 2, which utilizes a patch structure, behaves as a low-pass filter with a 3-dB cutoff frequency at 17 GHz, resulting in high $\:{S}_{21}$ of 7 dB at 28 GHz. Next, regarding angular stability under varying incidence angles, case 5 demonstrates the highest Q-factor^5,9^, leading to stability for $\:{\phi\:}^{TE}$ mode incidence angles up to 60$\:^\circ\:$, as shown in Fig. 3b. Finally, in terms of CRA, case 1 exhibits the lowest CRA at 1.75%, while case 5 has a slightly higher.

**Fig. 4:**
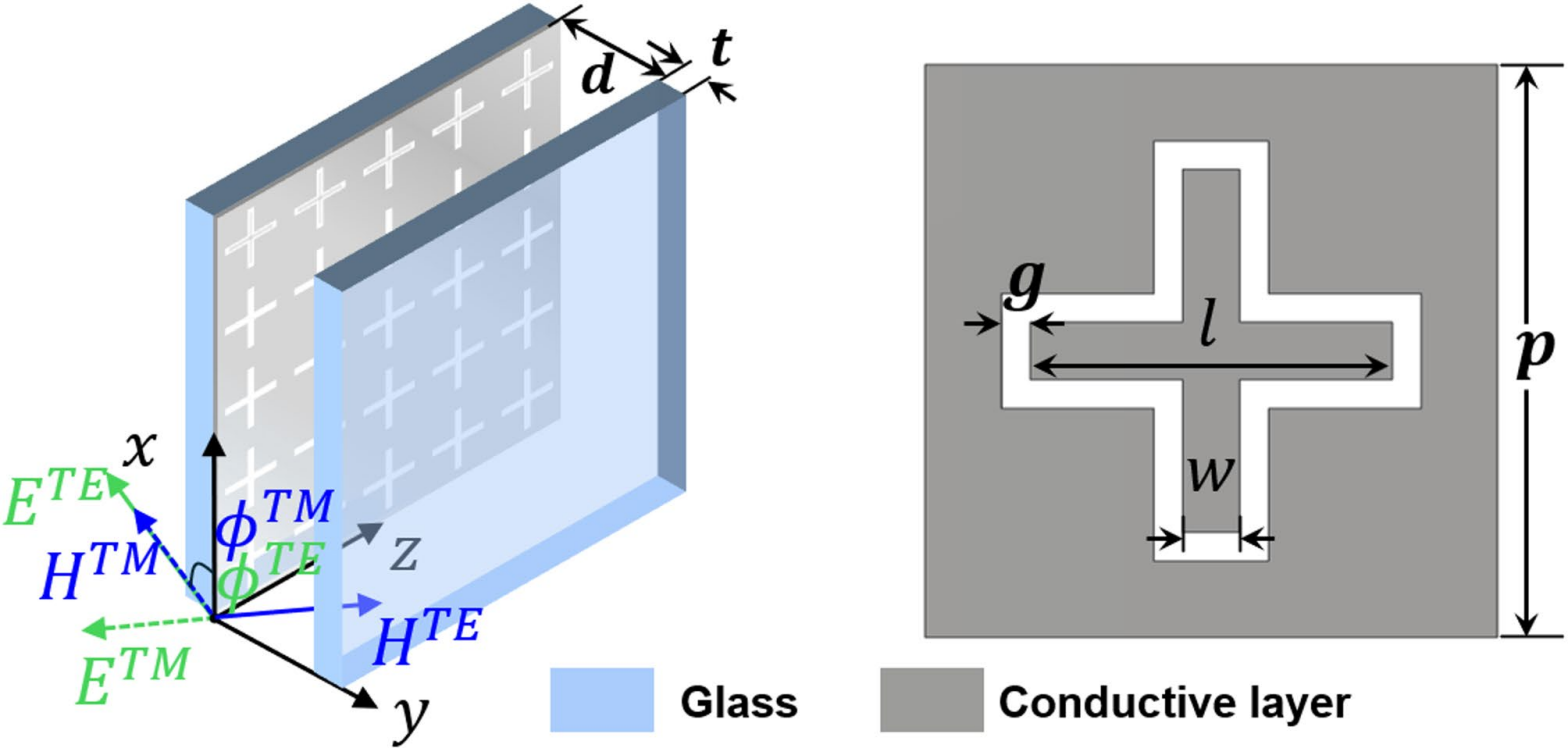
Schematic of the proposed double-pane GPTS and its unit-cell dimensions.

value of 2.6%. In contrast, cases 2 and 3 achieve the highest CRA, reaching about 10%. Considering these factors, the modified cross-dipole structure demonstrates superior performance compared to previously studied designs and has therefore been proposed for investigation with low-E coating in this study.

Figure 4 illustrates the schematic of the proposed double-pane GPTS and its unit-cell dimensions. The single-pane GPTS consists of a modified cross dipole, which is formed by a conductive layer on glass with a thickness of ($\:t$). The unit cell dimensions of the proposed FSS are based on case 5 and are presented in Table 1. By incorporating an additional glass layer in free space at a distance $\:d$, a double-pane GPTS is formed. The distance $\:d$ is determined based on half of the wavelength in free space at 28 GHz, following the Fabry–Pérot interferometer method^14^. To achieve resonance at 28 GHz with the double-pane GPTS, simulation and measurement results were investigated with $\:d$ varying between the two glass layers. There, the value $\:d$ of 5.8 mm exhibits the lowest peak transmission at 28 GHz, reaching 0.2 dB, and has been used in this study.

### Equivalent circuit model

**Fig. 5:**
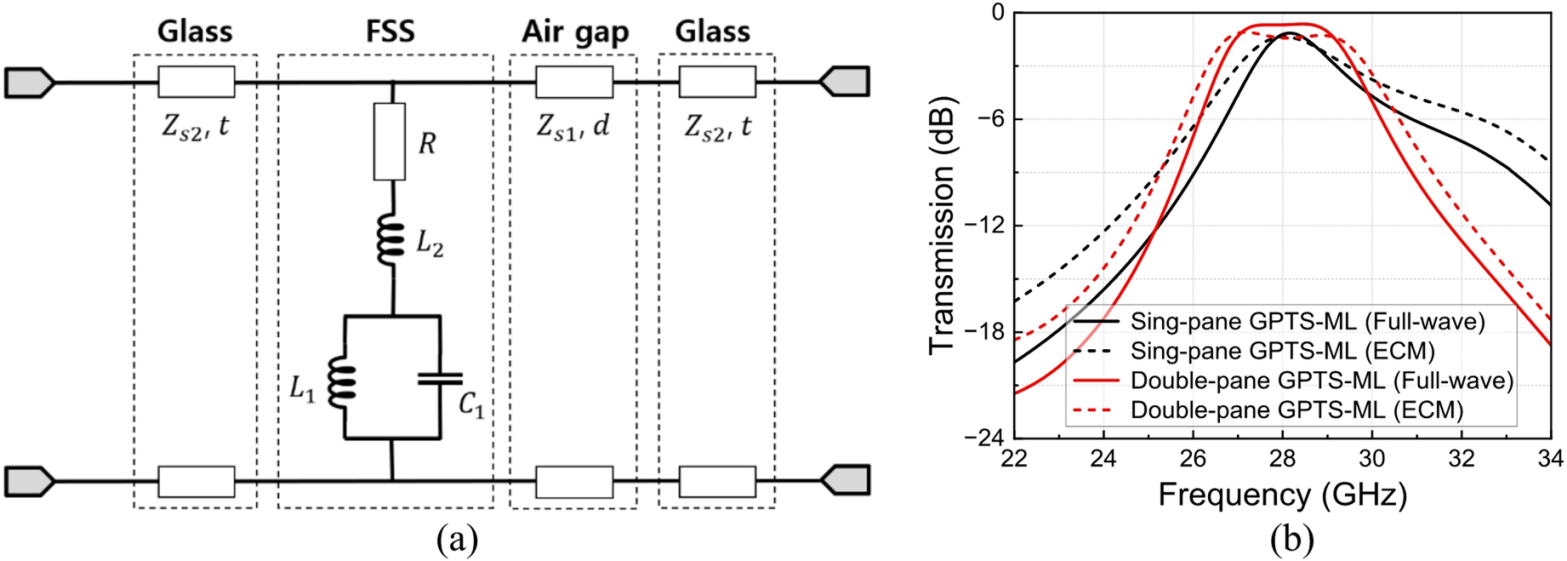
(**a**) Equivalent circuit model (ECM) of the proposed double-pane GPTS. (Parameters: $\:{L}_{1}$ = 0.037$\:nH$, $\:{L}_{2}$ = 0.086 $\:nH$, $\:{C}_{1}$ = 0.35 $\:pF$, $\:{Z}_{s1}$ = 377 Ω, and $\:{Z}_{s2}$ = 175.7 Ω). (**b**) Simulated transmission coefficients using full-wave EM and the ECM.

The ECM describes the operation of the proposed bandpass FSS based on EM field distributions. Figure 5a illustrates the formation of resistor ($\:R$), capacitance ($\:C$) and inductance ($\:L$) based on an incident $\:{\phi\:}^{TE}$-polarized wave parallel to the x-axis (see Fig. 4). The effective capacitance and inductance values can be calculated using^8^ for the ML case. The glass layer can be modeled by a short segment of a transmission line. By combining $\:R$, $\:L$ and $\:C$ elements within the unit cell structure, the impedance of the proposed FSS ($\:{Z}_{FSS}$) is calculated as Eq. (1):

1 $$\:{Z}_{FSS}=R+j\left(\omega\:{L}_{2}+\frac{\omega\:{L}_{1}}{1-{\omega\:}^{2}{L}_{1}{C}_{1}}\right)$$

where $\:R$ represents the lumped resistance of the proposed FSS. For the proposed FSS structures based on the ML, $\:R$ is typically very small. The resonant frequency of the proposed FSS can be determined using Eq. (1), and the Q-factor is given as Eq. (2):

2 $$\:Q=\frac{1}{R}\sqrt{\frac{{L}_{total}}{{C}_{total}}}$$

According to Eq. (2), this characteristic results in an increased Q-factor of case 5 compared to cases 1 to 4, leading to enhanced stability under varying incidence angles Fig. 3. Furthermore, the $\:{S}_{21}$ of the proposed FSS can be estimated using the ABCD transmission matrix^21^. The coefficients A, B, C, and D of the ABCD matrix for a system with multiple dielectric substrates can be expressed as Eqs. (3)–(5):

3 $$\:\left[\begin{array}{cc}A&\:B\\\:C&\:D\end{array}\right]=\left[{M}_{glass}\right]\left[{M}_{FSS}\right]\left[{M}_{air}\right]\left[{M}_{glass}\right]$$

4 $$\:\left[{M}_{FSS}\right]=\left[\begin{array}{cc}1&\:0\\\:1/{Z}_{FSS}&\:1\end{array}\right]$$

5 $$\:\left[{M}_{glass}\right]=\left[\begin{array}{cc}\mathrm{c}\mathrm{o}\mathrm{s}\left({k}_{z2}t\right)&\:\mathrm{j}{Z}_{1}\mathrm{s}\mathrm{i}\mathrm{n}\left({k}_{z2}t\right)\\\:j\frac{\mathrm{s}\mathrm{i}\mathrm{n}\left({k}_{z2}t\right)}{{Z}_{s2}}&\:\mathrm{c}\mathrm{o}\mathrm{s}\left({k}_{z2}t\right)\end{array}\right]$$

where $\:{k}_{z2}$ is the wavenumber of glass layer. The comparison of simulated transmission coefficients between the full-wave EM and ECM demonstrates high transmission responses for both single- and double-pane GPTSs, as shown in Fig. 5b. Both single- and double-pane GPTS-ML exhibit strong frequency responses at the resonant frequency of 28 GHz, with excellent agreement between the ECM and full-wave EM results. While both configurations provide high transmission, the double-pane GPTS-ML exhibits higher frequency selectivity than the single-pane GPTS-ML within the desired frequency and, therefore, offers uniformly low $\:{S}_{21}$ across a wide bandwidth.

### Discuss about coating layer

**Table 2:** Comparison with previous coating-layer approaches.

Low-E coating	Structure	OT (%)	$\:{\boldsymbol{R}}_{\boldsymbol{s}}$ (Ω/sq)	Haze (%)	Reflect-ance^*^ (*R*, %)	Flexi-bility	Fabrication Method
Polymer-metal hybrid electrode^20^ (This work)	PEI/Ag/PEDOT: PSS (PAP)	> 88 ($\:\sim$ 96 at 550 nm)	≤ 10	≤ 1	10–20	Yes ($\:r$ ≤ 5%)	Nano-nucleation + Evaporation
Transparent conducting oxides^12^^[,22^	ITO/FTO films	80–90	10–20	2–5	20–30	No	Sputtering + ALD
Ag-based Low-E ^7^ ^[,14^ ^[,15^	Glass/Ag-NW/Dielectric	80–90	≤ 10	N/A	50–80	Yes	Sputtering
DMD ^11^ ^[,22^	Dielectric/Metal-NW/Dielectric (DMD)	85–92	≤ 20	N/A	30–50	Yes	Sputtering
Multilayer coating ^9^	Metallic Oxide Layers + Metal-NW	$\:\sim$ 90	N/A	N/A	N/A	Yes	Sputtering

**Fig. 6:**
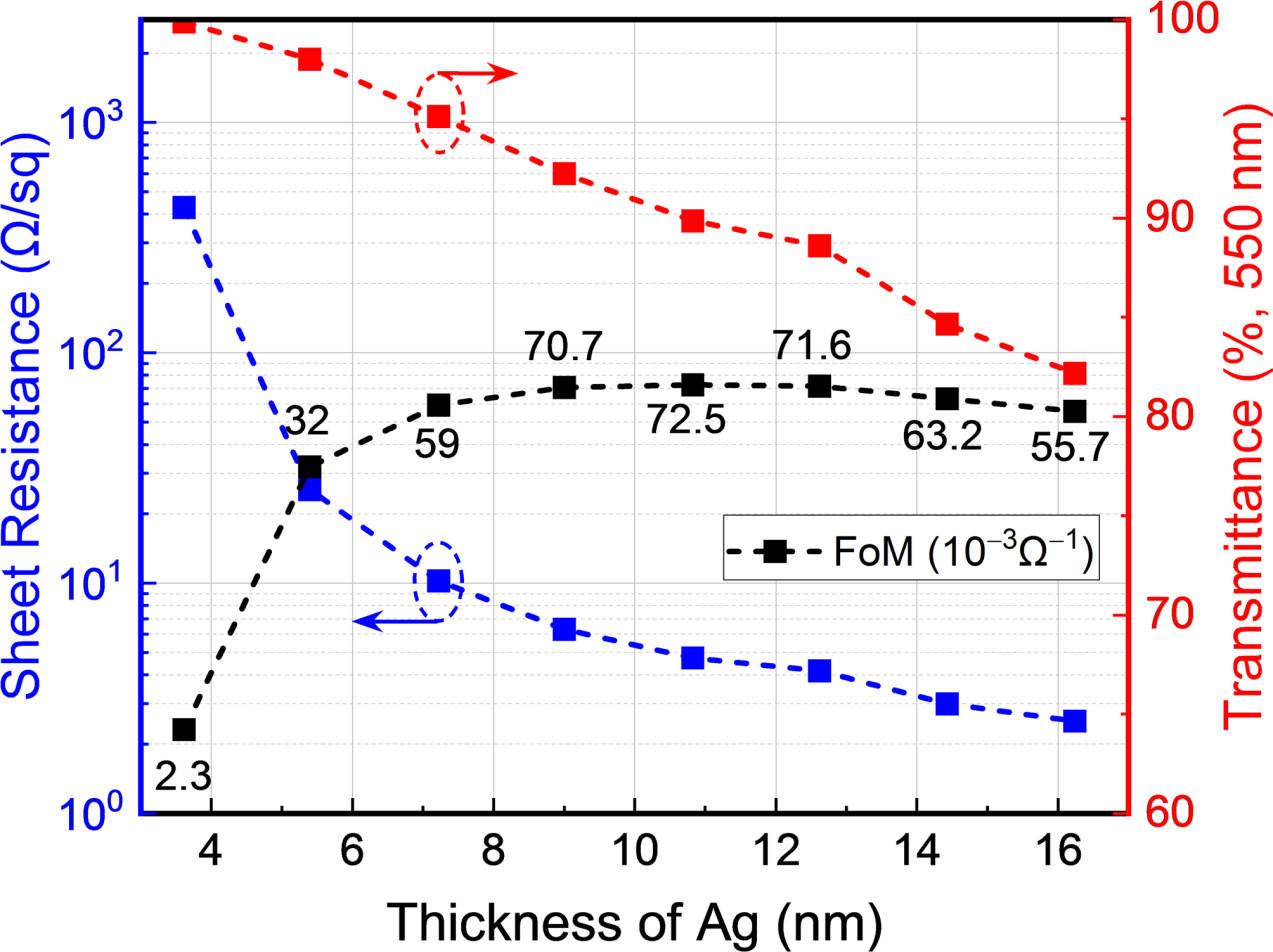
The calculated OT of the Ag coating is shown as a function of $\:{t}_{Ag}$ and $\:{R}_{s}$, where the values labeled along the black curve represent the corresponding FoM.

**Fig. 7:**
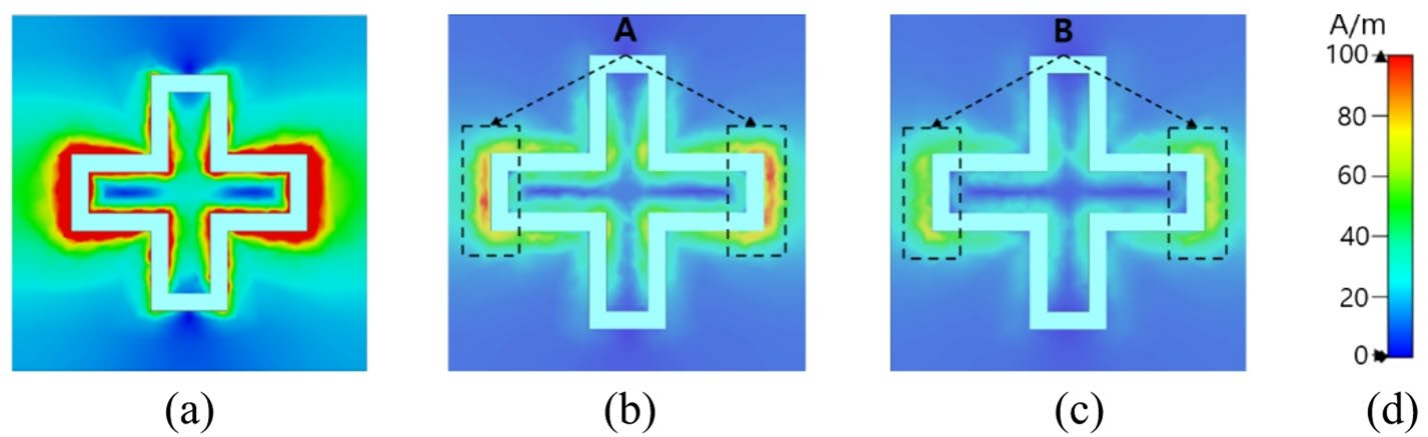
Surface current distribution on single-pane GPTS at 28 GHz ($\:{\phi\:}^{TE}$ mode) for different conductivities. (**a**) Copper. (**b**) LE film ($\:{R}_{s}=$ 5 Ω/sq**)**. (**c**) TE film ($\:{R}_{s}=$ 9.2 Ω/sq). (**d**) Magnitude.

In Table 2, the PEI/Ag/PEDOT: PSS (PAP) electrode, fabricated via metal-nucleation sites formation process with atomic-scale surface density, demonstrates superior performance compared to metal nanowires (NW). This improvement is attributed to the formation of a continuous and uniform silver (Ag) layer, which effectively eliminates the high interfacial resistance and light scattering typically observed in metal-NW structures. The PAP maintains a haze level of less than 1%, ensuring excellent optical transmittance (OT). It also achieves an average OT of 88% in visible light range, reaching 96% at 550 nm, along with low $\:{R}_{s}$ below 10 Ω/sq. Its the IR reflectance in the range of 10–20% enables efficient thermal regulation, comparable to that of the DMD structures, but with a more scalable thermal evaporation process. Additionally, thermal evaporation offers a lower-cost fabrication method compared to deposition and sputtering techniques.

Both the TE and LE coatings, based on the PAP structure, were deposited on a polyethylene terephthalate (PET) dielectric substrate with a thickness of approximately 50 μm. The PEI/Ag/PEDOT: PSS (PAP) refers to an electrode structure consisting of an Ag layer sandwiched between two distinct polymers—polyethyleneimine (PEI) and poly (3,4-ethylenedio xythiophene): poly(styrenesulfonate) (PEDOT: PSS)^20,23^.

In this work, the $\:{R}_{s}$ of the TE and LE coatings are 9.2 Ω/sq and 5 Ω/sq, respectively, with corresponding thicknesses of 127 μm and 67 μm. These values were provided by MSWAY; however, detailed information regarding the electrodes (Ag, PEI, and PEDOT: PSS) has remained confidential due to commercial restrictions. Nevertheless, the relationship between thickness of Ag ($\:{t}_{Ag}$), $\:{R}_{s}$, and optical transmittance at 550 nm ($\:T,\%$​) can be estimated as Eq. (6)^24^:

6 $$\:T(\%,\:\:500\:\mathrm{n}\mathrm{m})={\left(1+\frac{60\pi\:}{{R}_{s}}\frac{{\sigma\:}_{op}}{{\sigma\:}_{dc}}\right)}^{-2}$$

where $\:{\sigma\:}_{dc}$​ (S/m) denotes the DC conductivity, and $\:{\sigma\:}_{op}$​ (S/m) represents the optical conductivity. It is assumed that the ratio $\:{\sigma\:}_{op}/{\sigma\:}_{dc}$ remains constant for varying $\:{t}_{Ag}$within the optical frequency range^25^. Figure 6 shows that the OT of the Ag coating can be estimated using Eq. (6), with $\:{R}_{s}$​ values measured^20^, and the performance of transparent conductors can be further evaluated using the figure of merit (FoM), defined as: FoM ($\:{10}^{-3}{{\Omega\:}}^{-1})={T}^{10}/{R}_{s}$.

The resistance of the FSS for the coated case can be estimated based on the $\:{R}_{s}$, the coating-covered area within the unit cell ($\:{A}_{coating})$, the total unit cell area ($\:{A}_{unitcell})$, and fill ratio ($\:F$) as Eq. (7):

7 $$\:R={R}_{s}\frac{{A}_{unitcell}}{{A}_{coating}}=\frac{{R}_{s}}{F}$$

Figure 7 shows the current distribution at 28 GHz on the conductive layer for increasing $\:{R}_{s}$, including copper, LE ($\:{R}_{s}=$ 5 Ω/sq), and TE ($\:{R}_{s}=$ 9.2 Ω/sq). In^5^, the dissipated power​ is directly related to the complex impedance of the FSS elements, as given in Eq. (1). According to Eq. (7), for a given frequency, an increase $\:R$, or a decrease $\:F$, results in higher power dissipation, leading to reduced transmission. When comparing FSS structures with equal $\:F$, the circular and square patch elements exhibit a more distributed effective current area, thereby yielding lower current density. In contrast, the cross-dipole structure concentrates the surface current along narrow branches, as highlighted in regions A and B of Fig. 7b and Fig. 7c, respectively. This localized current concentration enhances the resonant response at the transmission frequency, thereby improving transmission performance.

### Measurement method of the GPTSs

**Fig. 8:**
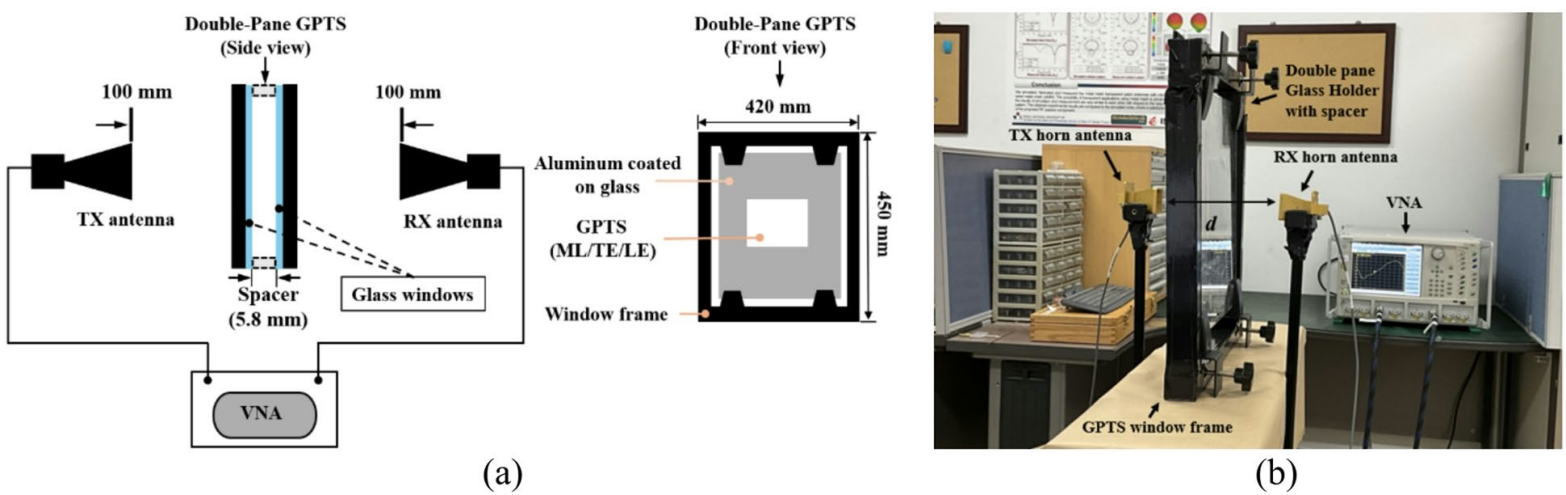
Measurement setup for transmission characteristics of double-pane GPTS. (**a**) Block diagram. (**b**) Photograph.

Figure 8a illustrates the block diagram of the measurement setup for the double-pane GPTS prototypes (GPTS-ML, GPTS-TE, GPTS-LE). Two horn antennas—one for transmitter (TX) and one for receiver (RX)—were mounted on tripods and connected to a vector network analyzer (Anritsu MS4644B) operating from 10 GHz to 40 GHz. The antennas were placed 100 mm apart in the far-field region from the metal surface, satisfying the far-field condition defined by 2D²/λ. To experimentally evaluate the proposed FSS performance, the ML sample with dimensions of 15 × 15 cm² was fabricated using a screen-printing process (Pil Solution Co., Ltd., Korea). The TE and the LE samples, each with dimensions of 12 × 12 cm², were fabricated by laser ablation to form the slot gaps and were supplied by MSWAY. In addition, the transparent adhesive used has a thickness of 25 μm and an excellent OT of up to 99%, as provided by 3 M Co^26^. The.

double-pane GPTS samples were mounted on a wooden frame with spacers to maintain the gap between the glass panes. The frame measured 420 mm × 450 mm with a 350 mm × 350 mm window to hold the FSS prototypes. To minimize multipath reflections and diffraction, aluminum foil was applied to one side of the frame, leaving a square opening at the center matching the FSS prototype area. Figure 8b shows a photograph of the measurement setup used for testing the double-pane GPTS. During the measurement process, the two horn antennas were perpendicular to the plane surface of the GPTS for normal incidence. Additional measurements were performed at incidence angles of 30°, 45°, and 60° for both TE and TM polarizations.

### Results of the GPTS-ML

**Fig. 9:**
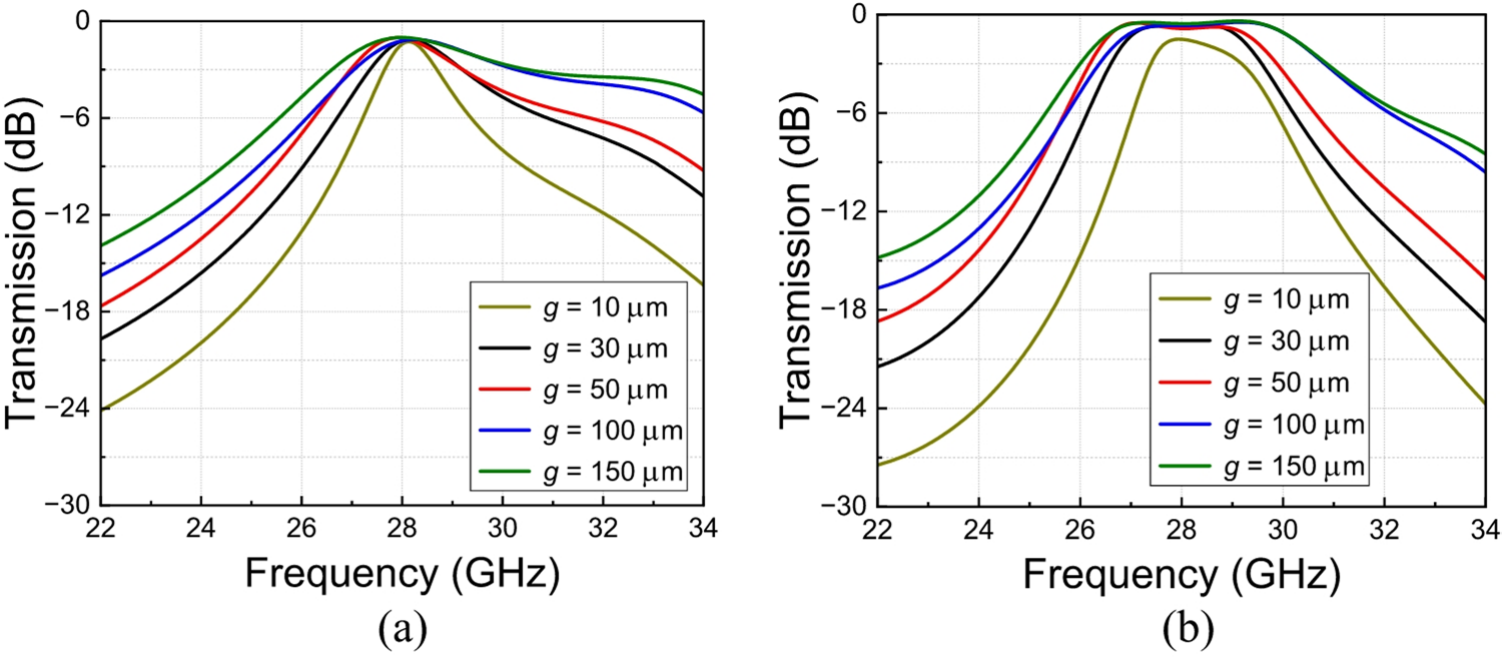
Simulated transmission coefficients for different slot gap variations. (**a**) Single-pane GPTS-ML. (**b**) Double-pane GPTS-ML.

Figure 9a shows the simulated transmission coefficients of a single-pane GPTS-ML for different values of $\:g$. The bandpass filter achieves a center frequency of 28 GHz for each value of $\:g$. As $\:g$ decreases, out-of-band rejection improves, and the Q-factor increases. Figure 9b presents the simulated results for the double-pane GPTS-ML structure with a fixed interlayer spacing $\:d$ of 5.8 mm and $\:g$ is varied. With the addition of a second glass layer, the structure behaves like a Fabry–Pérot resonator, introducing additional intrinsic resonances and enhancing the resonance effect, which results in improved frequency selectivity. Accordingly, a gap width of $\:g$ = 30 μm was identified as the optimal value for both single- and double-pane GPTS-ML structures and was therefore selected for fabrication.

**Fig. 10:**
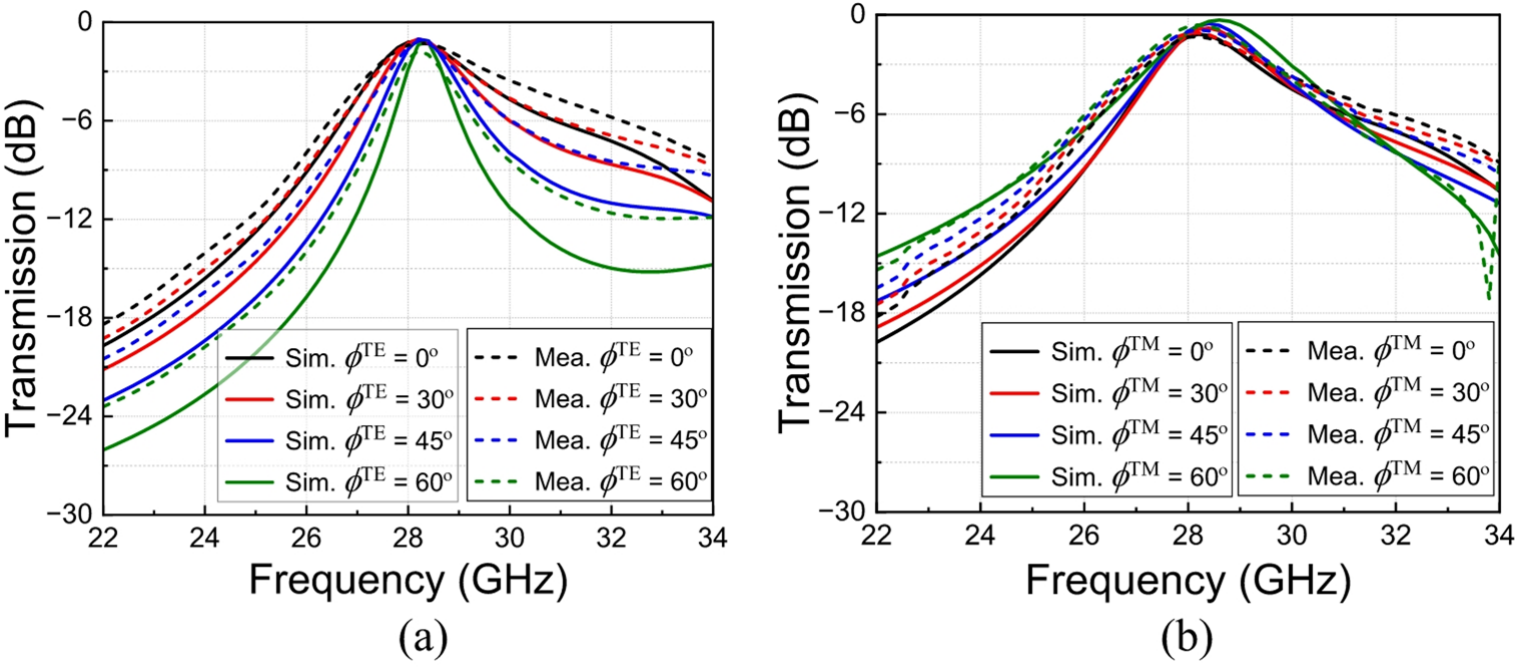
Simulation and measurement results of $\:{S}_{21}$ for single-pane GPTS-ML at various incidence angles. (**a**) $\:{\phi\:}^{TE}$ mode. (**b**) $\:{\phi\:}^{TM}$ mode.

Figure 10 shows the simulated and measured transmission coefficients of the single-pane GPTS-ML for both $\:{\phi\:}^{TE}$ and $\:{\phi\:}^{TM}$ mode at incidence angles of 0°, 30°, 45°, and 60°. Good agreement is observed between.

**Fig. 11:**
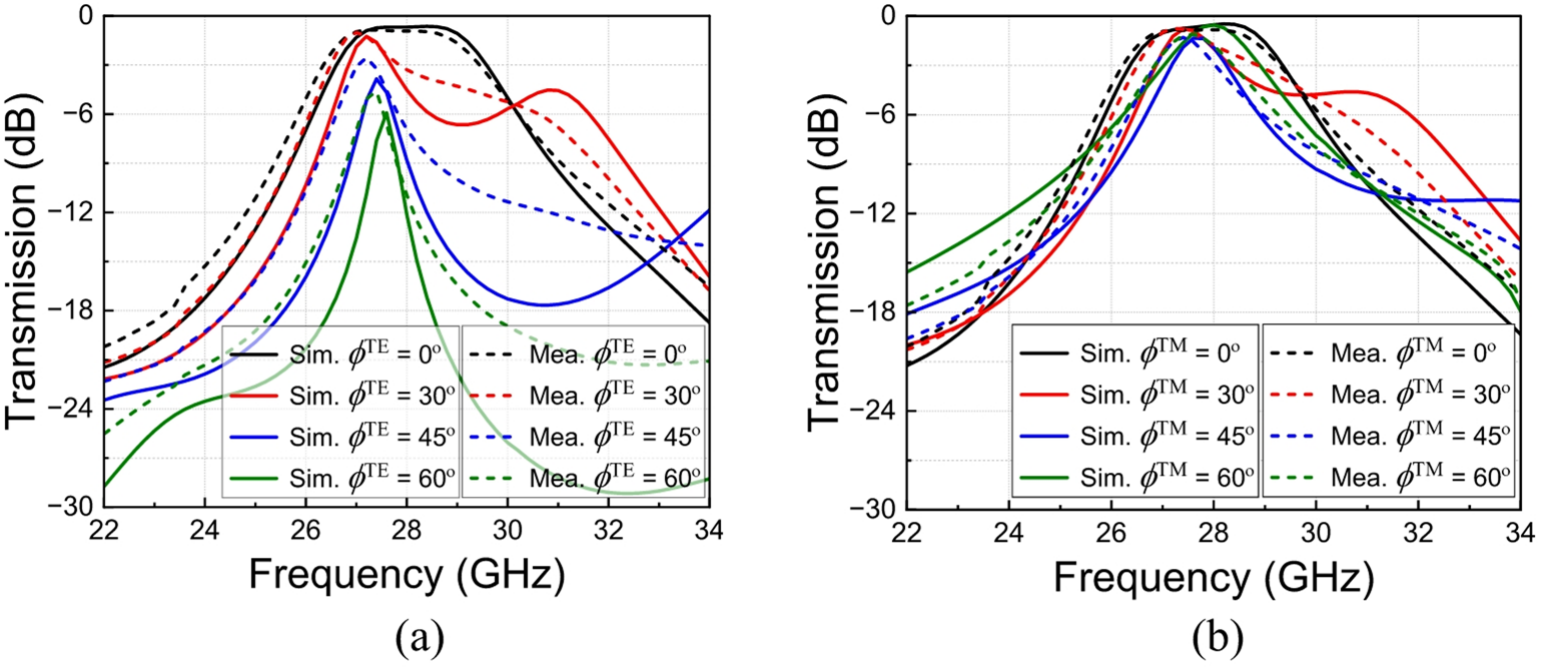
Simulation and measurement results of $\:{S}_{21}$ for double-pane GPTS-ML at various incidence angles. (**a**) $\:{\phi\:}^{TE}$ mode. (**b**) $\:{\phi\:}^{TM}$ mode.

the simulated and measured results. For the $\:{\phi\:}^{TE}$ mode [Fig. 10a], as the incidence angle increases, the resonance frequency shifts slightly toward higher frequencies, with an average $\:{S}_{21}$ of approximately 1.6 dB at 28 GHz. For the $\:{\phi\:}^{TM}$ mode [Fig. 10b], the resonance frequency remains nearly constant, and the average $\:{S}_{21}$ is approximately 1.05 dB at 28 GHz. These results confirm that the $\:F$ modified cross-dipole structure maintains stable transmission at incidence angles up to 60° for both $\:{\phi\:}^{TE}$ and $\:{\phi\:}^{TM}$ modes under ideal conductivity conditions.

Figure 11 presents both simulation and measurement results of the transmission coefficient for the double-pane GPTS-ML structure under various incidence angles in both $\:{\phi\:}^{TE}$ and $\:{\phi\:}^{TM}$ polarization modes. As the $\:{\phi\:}^{TE}$-incidence angle increases from 0° to 60°, a clear shift in the resonance frequency and degradation in transmission amplitude are observed. In this case, the resonance peak significantly shifts toward higher frequencies, and the transmission amplitude decreases, indicating a degradation in angular stability. This behavior can be explained using the Fabry–Pérot resonance in Eq. (8)^27^:

8 $$\:{f}_{res}=\frac{mc}{2ndcos\phi\:}$$

where $\:{f}_{res}$​ is the resonance frequency, $\:c\:$is the speed of light in vacuum, $\:n$ is the refractive index of the glass, and $\:\phi\:$ is the transmitted angle within the dielectric. As the angle incidence of $\:{\phi\:}^{TE}$ mode increases, the refraction angle $\:\phi\:$ increases due to Snell’s Law, which shifts the $\:{f}_{res}$​ upward. In contrast, the $\:{\phi\:}^{TM}$ mode exhibits better angular stability, with only slight resonance shifts and relatively consistent transmission characteristics across varying angles.

## Double-pane GPTS with transparent electrode (GPTS-TE)

**Fig. 12:**
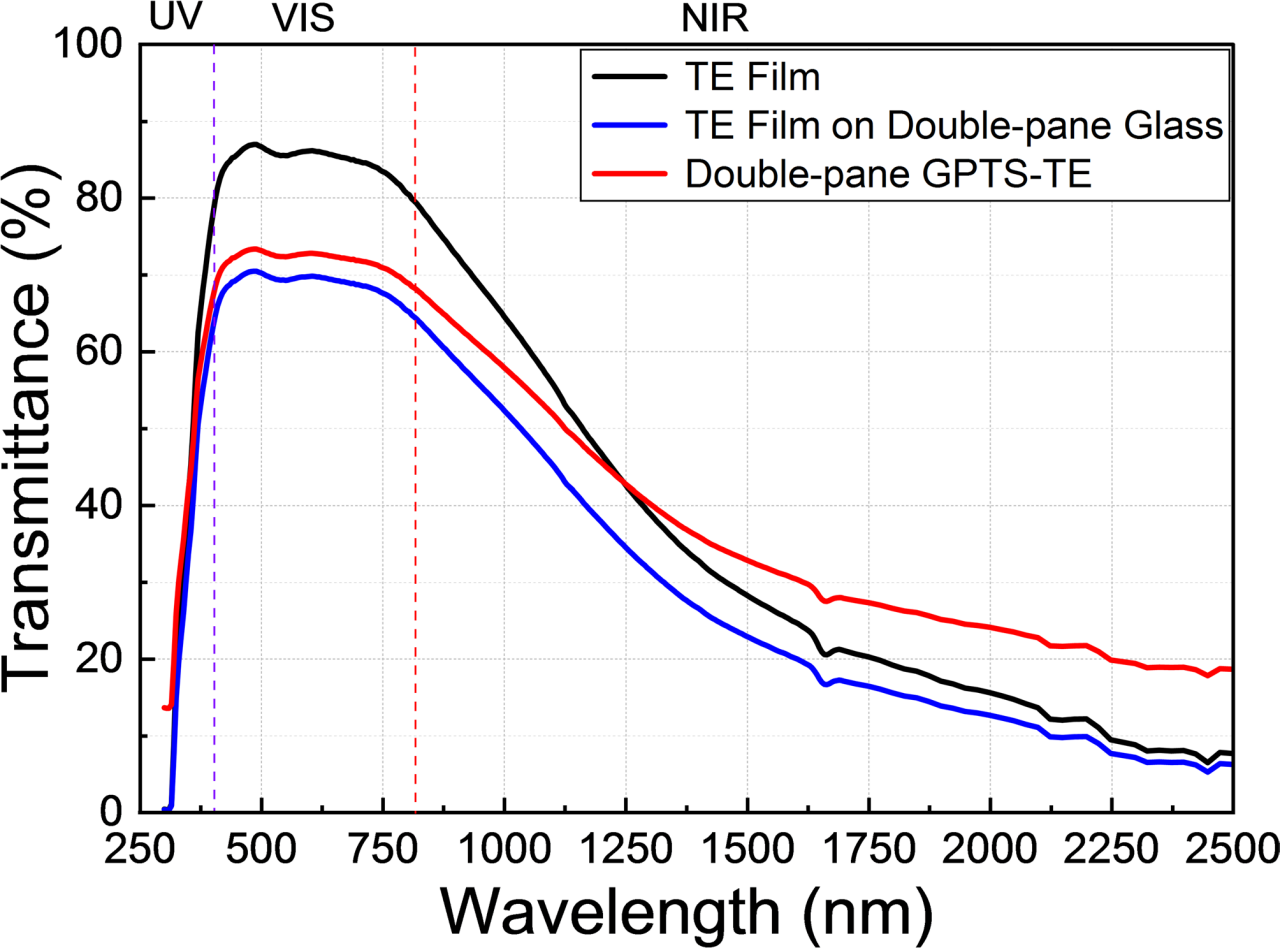
Measured transmittance of the TE film, the TE film on double-pane glass, and the double-pane GPTS-TE structure over wavelength ranges corresponding to UV (250–380 nm), VIS (380–780 nm), and NIR (780–2500 nm).

After determining the optimal FSS structure, the ML was replaced with a TE configuration to investigate its transmission and multispectral compatibility, aiming at enhancing transmission through double-pane low-E windows. The TE film has a $\:{R}_{s}$ of 9.2 Ω/sq and a film thickness of 127 μm. To improve the $\:{T}_{\:vis}$ of the double-pane GPTS-TE and enhance transmission at 28 GHz, the slot gap $\:g$ was optimized to 150 μm and the dipole length $\:l$ to 1.94 mm, yielding a fill ratio $\:F$ of 0.86. The $\:{T}_{\:vis}$ of the double-pane GPTS-TE structure is evaluated in Eq. (9):

9 $$\:{T}_{\:vis}={T}_{vis-glass}^{2}\left(1-F\right)+{T}_{vis-coating}{T}_{vis-glass}^{2}F$$

where $\:{T}_{\:vis-glass}$ denotes the optical transmittance of the glass substrate, and $\:{T}_{vis-coating}$ refers to the optical transmittance of the TE film. Based on the measurement results, the $\:{T}_{\:vis-glass}$ and the $\:{T}_{vis-coating}$ in the visible light band are 90% and 80%, respectively. Therefore, in Eq. (9), the $\:{T}_{\:vis}$ of GPTS-TE can be calculated to be approximately 67%.

Figure 12 shows the measured transmittance of the TE film, the TE film on double-pane glass, and the double-pane GPTS-TE structure over wavelength ranges from 250 nm to 2500 nm. The TE film achieved an average $\:{T}_{\:vis}$ of 80% and 47% in IR transmittance ($\:{T}_{IR}$). When combining TE film with double-pane glass, the $\:{T}_{\:vis}$ and the $\:{T}_{\:IR}$ decreased to around 64% and 38%, respectively. In the double-pane GPTS-TE, the FSS patterning reduced the CRA by 14.8%, resulting in a slight increase in the $\:{T}_{\:IR}$ of 44%, while maintaining the $\:{T}_{\:vis}$ at 67%. This demonstrates that compared to double-pane glass with an average transmittance of 81% for both the $\:{T}_{\:vis}$ and the $\:{T}_{\:IR}$, the double-pane GPTS-TE design effectively blocks IR waves while maintaining transparency in visible light.

**Fig. 13:**
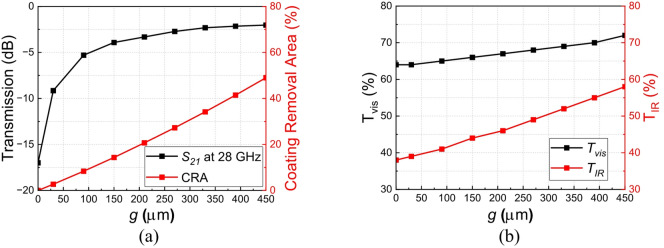
Effect of slot gap $\:g$ on the multispectral compatibility of the double-pane GPTS-TE. (**a**) Simulated transmission $\:{S}_{21}$ at 28 GHz and the corresponding CRA. (**b**) Calculated $\:{T}_{\:vis}$ and $\:{T}_{\:IR}$.

Figure 13 illustrates the transmission and multispectral compatibility of the double-pane GPTS-TE structure as a function of $\:g$. In Fig. 13a, the simulated $\:{S}_{21}$ at 28 GHz significantly improves with increasing $\:g$, while the CRA increases linearly. Figure 13b, based on Eq. (9), shows that $\:{T}_{\:vis}$ increases slightly by approximately 9%, whereas $\:{T}_{\:IR}$ shows a more substantial rise of about 22%. At $\:g=$ 90 μm, $\:{S}_{21}$ reaches 5.3 dB, with a low CRA of 8.3%, $\:{T}_{\:vis}$ of 65%, and $\:{T}_{\:IR}$​ of 41%, indicating a potentially optimal trade-off. At $\:g=$ 150 μm, $\:{S}_{21}$ improves to 4 dB, CRA increases to 14.3%, with $\:{T}_{\:vis}$​ of 66% and $\:{T}_{\:IR}$​ of 44%. However, to optimize fabrication cost, a gap width of 150 μm for $\:g$ was selected.

**Fig. 14:**
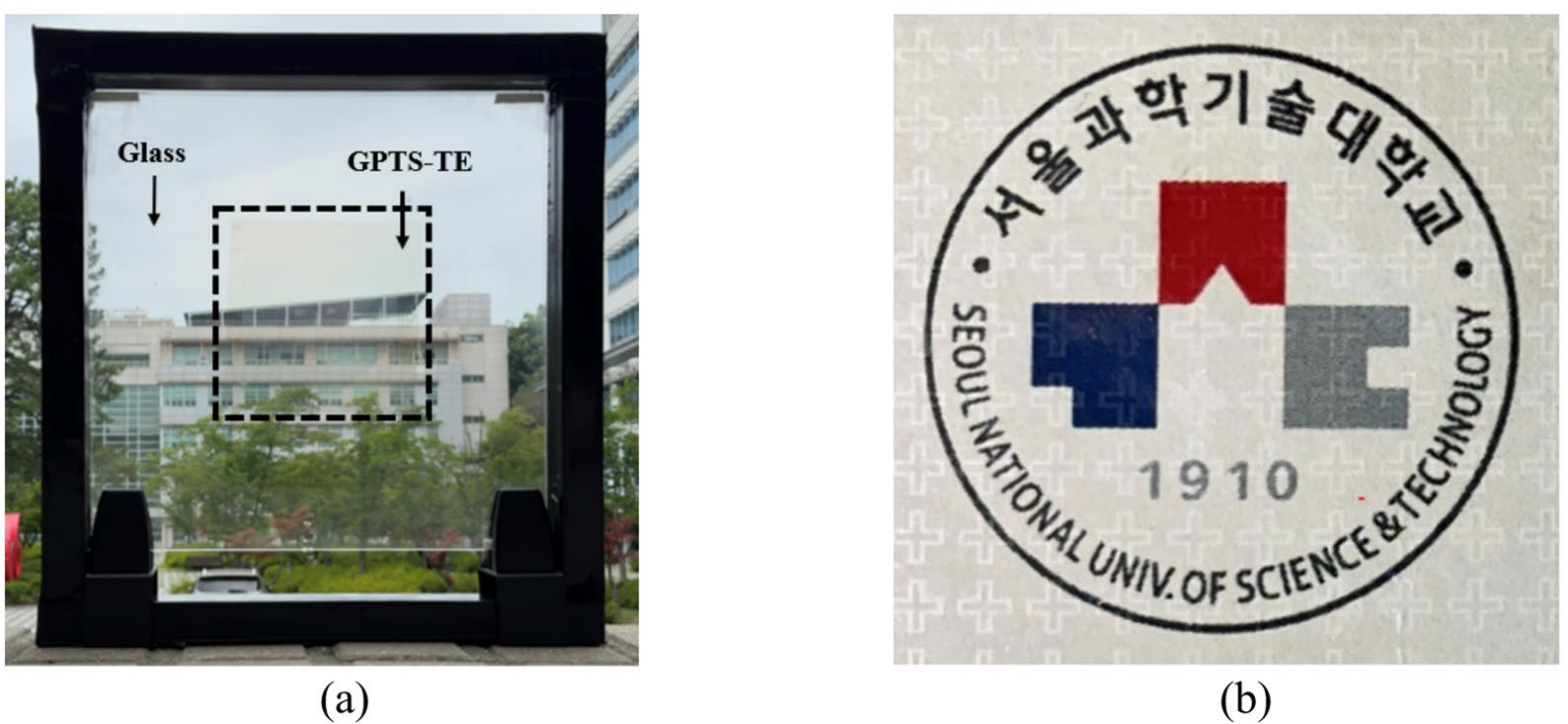
(**a**) Photograph of the GPTS-TE sample with a size of 12 × 12 cm². (**b**) Photograph of the TE film with FSS patterns placed over the university logo.

Figure 14a shows an outdoor photograph of the double-pane glass window prototype, in which the GPTS-TE region (indicated by the dashed square) is arranged in the same manner as the proposed structure in Fig. 4. Figure 14b illustrates the fabrication process of the TE-coated sample with an FSS pattern, which is difficult to distinguish from the naked eye. Although there is a slight reduction in optical transparency between the un-patterned double-pane glass and the fabricated GPTS-TE, the structure remains aesthetically transparent.

**Fig. 15:**
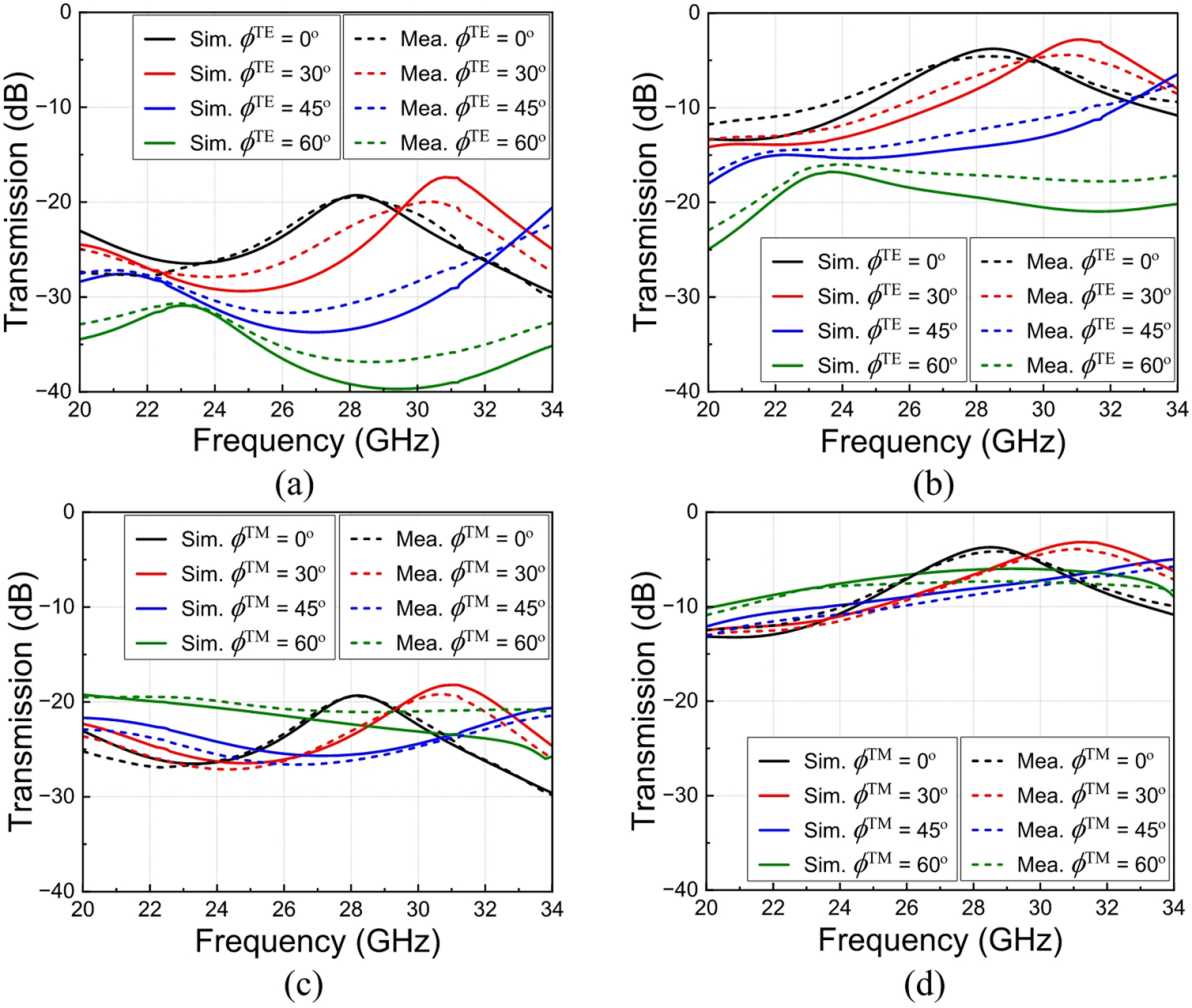
Simulation and measurement results of $\:{S}_{21}$ with varying incidence angles. (**a**) TE film on double-pane glass at $\:{\phi\:}^{TE}$ mode. (**b**) Double-pane GPTS-TE at $\:{\phi\:}^{TE}$ mode. (**c**) TE film on double-pane glass at $\:{\phi\:}^{TM}$ mode. (d) Double-pane GPTS-TE at $\:{\phi\:}^{TM}$ mode.

Figure 15 presents the simulated and measured transmission results at various incidence angles under both $\:{\phi\:}^{TE}$ and $\:{\phi\:}^{TM}$ polarizations. The measured transmission coefficients at 28 GHz are summarized in Table 3. Figure 15a shows the performance of the TE film fully coated on the double-pane glass structure, where the transmission decreases significantly as the incidence angle increases. In the $\:{\phi\:}^{TE}$ mode, the tangential component of the electric field increases with the incidence angle, generating stronger surface currents on the conductive coating^4^. This results in higher ohmic losses due to the $\:{R}_{s}$ of the coating, leading to a significant decrease in transmission at oblique angles. For the $\:{\phi\:}^{TE}$ mode at 28 GHz, compared with the TE film on double-pane glass, the double-pane GPTS-TE shows improvements of 14.8 dB, 16 dB, 18.4 dB, and 10.6 dB at incidence angles of 0°, 30°, 45°, and 60°, respectively. In contrast, the $\:{\phi\:}^{TM}$ mode exhibits a weaker tangential electric field, leading to lower surface currents and reduced loss. In Figs. 15c and 15 d, the GPTS-TE structure shows $\:{S}_{21}$ improvements of 15.04 dB, 16.3 dB, 17.4 dB, and 13.7 dB over the fully coated case at the corresponding incidence angles of 0°, 30°, 45°, and 60°.

**Table 3:** Measured results of $\:{S}_{21}$ at 28 GHz for the TE film on double-pane glass and the double-pane GPTS-TE.

Structure	$\:{\boldsymbol{S}}_{21}$ (dB) for $\:{\boldsymbol{\phi\:}}^{\boldsymbol{T}\boldsymbol{E}}$ mode				$\:{\boldsymbol{S}}_{21}$ (dB) for $\:{\boldsymbol{\phi\:}}^{\boldsymbol{T}\boldsymbol{M}}$ mode			
	0°	30°	45°	60°	0°	30°	45°	60°
TE film on double-pane glass	19.5	22.6	30.7	27.7	19.34	23	26.1	21
Double-pane GPTS-TE	4.7	6.6	12.3	17.1	4.3	6.7	8.7	7.3

## Double-pane GPTS with low-e coating (GPTS-LE)

To specifically block IR radiation, the double-pane GPTS-LE is designed using a low-E film composed of a thin metallic layer and multiple dielectric material. The LE film has a $\:{R}_{s}$ of 5 Ω/sq and a film thickness of 67 μm. The design and fabrication follow the same process as the GPTS-TE.

**Fig. 16:**
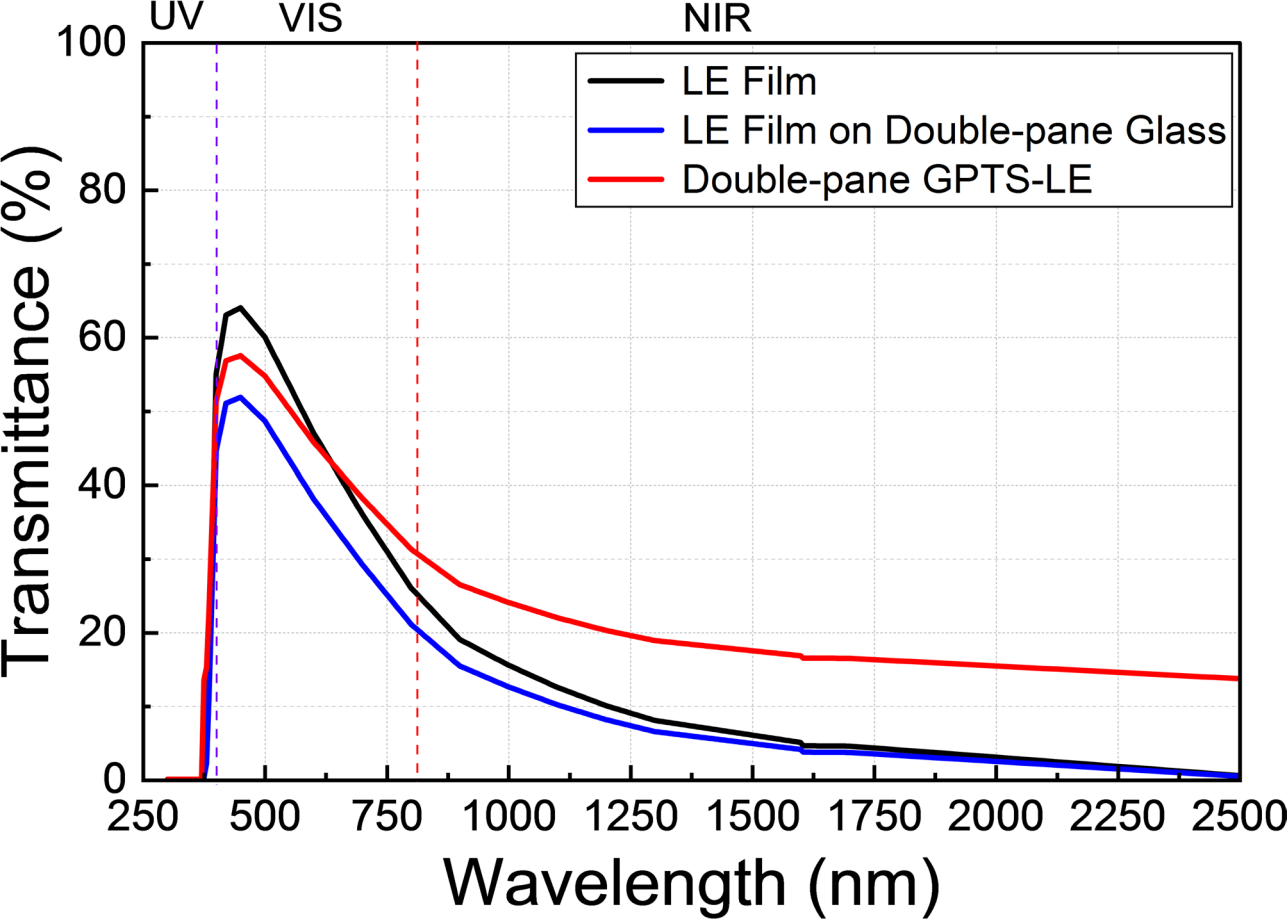
Measured transmittance of the LE film, the LE film on double-pane glass, and the double-pane GPTS-LE structure over wavelength ranges corresponding to UV (250–380 nm), VIS (380–780 nm), and NIR (780–2500 nm).

Figure 16 shows the measured transmittance of the LE film, the LE film on double-pane glass, and the double-pane GPTS-LE structure over wavelength ranges from 250 nm to 2500 nm. The LE film demonstrated an average $\:{T}_{\:vis}$ of 41% and 11% for $\:{T}_{\:IR}$. When combined with the double-pane glass, considering the glass’s transmittance, the $\:{T}_{\:vis}$ and $\:{T}_{\:IR}$ decreased to 33% and 9%, respectively. For the double-pane GPTS-LE, the FSS patterning reduced the coated area by 14.8%, leading to a significant.

**Fig. 17:**
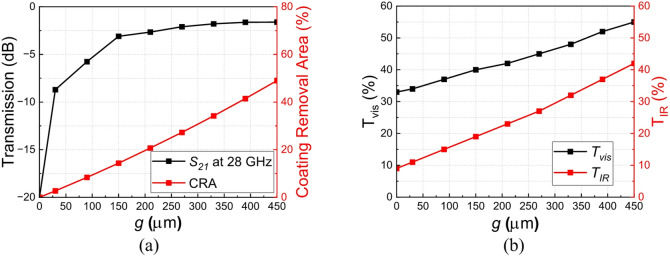
Effect of slot gap $\:g$ on the multispectral compatibility of the double-pane GPTS-LE. (**a**) Simulated transmission $\:{S}_{21}$ at 28 GHz and the corresponding CRA. (**b**) Calculated $\:{T}_{\:vis}$ and $\:{T}_{\:IR}$.

increase in $\:{T}_{\:IR}$ to 19%, with only a slight change in $\:{T}_{\:vis}$, which reached 40%. This GPTS-LE design effectively blocks IR waves while maintaining transparency in visible light.

The effect of $\:g$ on the transmission and multispectral compatibility of the double-pane GPTS-LE is shown in Fig. 17. When $\:g$ increases from 0 to 150 μm, the $\:{S}_{21}$ at 28 GHz improves significantly from 20 dB to 3.5 dB. Beyond 150 μm, the additional improvement becomes insignificant, with $\:{S}_{21}$ decreasing only from 3.5 dB to 1.8 dB at 450 μm. Meanwhile, as $\:g$ increases, the CRA also grows substantially, leading to an increase in $\:{T}_{\:vis}$ from 34% to 55% and $\:{T}_{\:\mathrm{I}\mathrm{R}}$ from 9% to 42%. This indicates a clear trade-off between transmission and multispectral compatibility. Therefore, the slot gap $\:g$ was set to 150 μm for the double-pane GPTS-LE, as it provides an optimal balance between transmission and multispectral compatibility in this study.

**Fig. 18:**
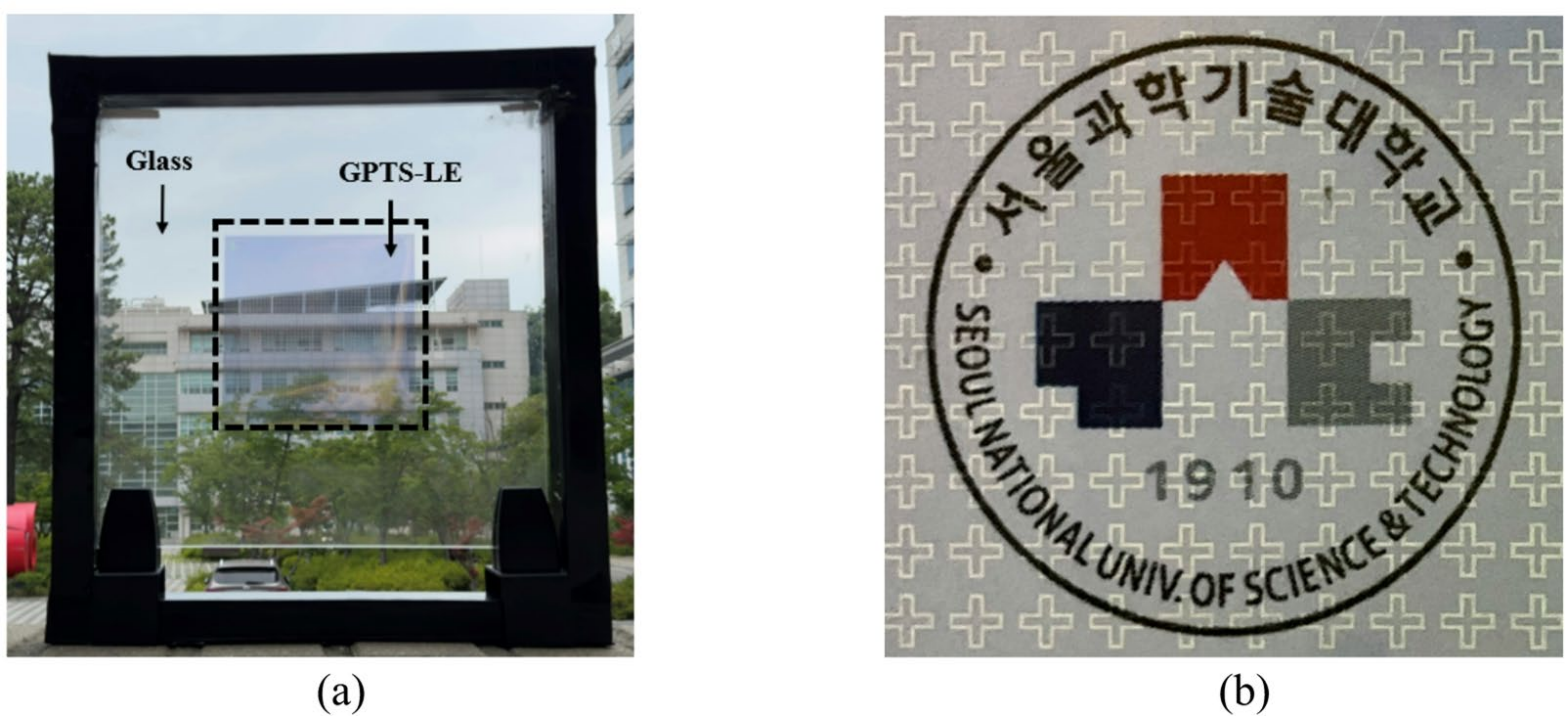
(**a**) Photograph of the GPTS-LE sample with a size of 12 × 12 cm². (**b**) Photograph of the LE film with FSS patterns placed over the university logo.

Figure 18a shows an outdoor photograph comparing the visible transparency of the glass and the GPTS-LE region (indicated by the dashed square). Compared to the double-pane GPTS-TE, the GPTS-LE prototype exhibits lower OT but offers significant advantages due to its excellent IR-blocking capability. Figure 18b shows the fabricated LE-coated sample placed over the university logo. Compared with the TE-coated sample, the LE-coated region exhibits a slightly different color. This difference arises from the different $\:{R}_{s}$ of the TE and LE films, since the higher $\:{R}_{s}$ of the TE film corresponds to a thinner Ag layer with higher OT, whereas the lower $\:{R}_{s}$ of the LE film corresponds to a thicker Ag layer, resulting in a darker.

**Fig. 19:**
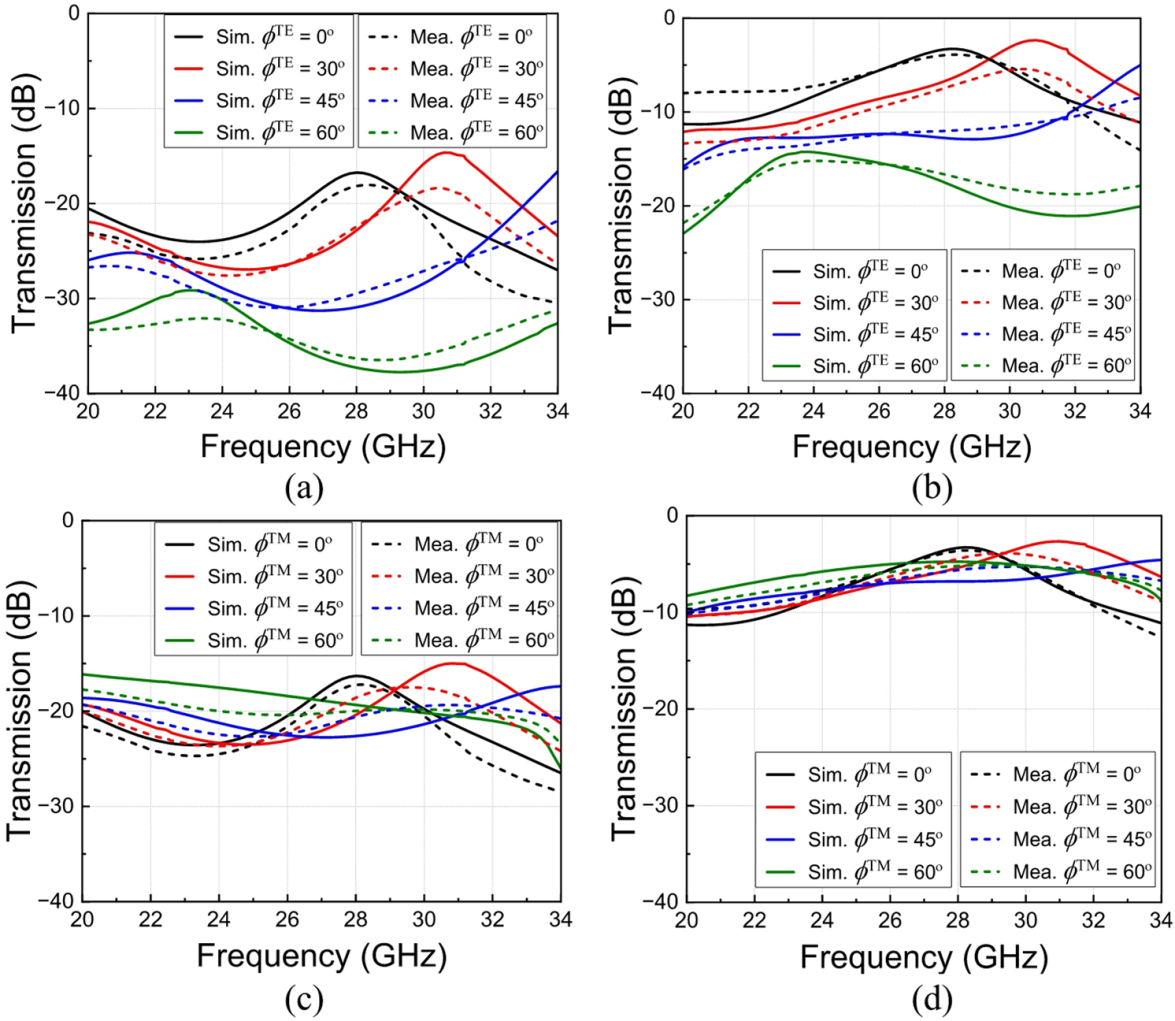
Simulation and measurement results of $\:{S}_{21}$ with varying incidence angles. (**a**) LE film on double-pane glass at $\:{\phi\:}^{TE}$ mode. (**b**) Double-pane GPTS-LE at $\:{\phi\:}^{TE}$ mode. (**c**) LE film on double-pane glass at $\:{\phi\:}^{TM}$ mode. (**d**) Double-pane GPTS-LE at $\:{\phi\:}^{TM}$ mode.

Figure 19 shows the simulated and measured transmission performance under varying incidence angles for both $\:{\phi\:}^{TE}$ and $\:{\phi\:}^{TM}$ modes using LE film on double-pane glass and the double-pane GPTS-LE. There is good agreement between the simulation and measurement results. The measured results of $\:{S}_{21}$ at 28 GHz are summarized in Table 4. For $\:{\phi\:}^{TE}$ mode at 28 GHz, compared to the fully coated LE film, the double- pane GPTS-LE achieves $\:{S}_{21}$ improvements of 14.2 dB, 14.9 dB, 17.4 dB, and 19.6 dB at incidence angles of 0°, 30°, 45°, and 60°, respectively. For $\:{\phi\:}^{TM}$ mode at 28 GHz, the GPTS-LE structure shows $\:{S}_{21}$ improvements of 13.6 dB, 14.1 dB, 14.9 dB, and 14.7 dB over the fully coated case at the corresponding incidence angles of 0°, 30°, 45°, and 60°.

**Table 4:** Measured results of $\:{S}_{21}$ at 28 GHz for the LE film on double-pane glass and the double-pane GPTS-LE.

Structure	$\:{\boldsymbol{S}}_{21}$ (dB) for $\:{\boldsymbol{\phi\:}}^{\boldsymbol{T}\boldsymbol{E}}$ mode				$\:{\boldsymbol{S}}_{21}$ (dB) for $\:{\boldsymbol{\phi\:}}^{\boldsymbol{T}\boldsymbol{M}}$ mode			
	0°	30°	45°	60°	0°	30°	45°	60°
LE film on double-pane glass	18.2	22.3	29.4	36.2	17.2	18.6	20.5	19.8
Double-pane GPTS-LE	4	7.4	12	16.6	3.6	4.5	5.6	5.1

## Comparison and discussion

**Table 5:** Comparison with previous works.

Ref.	Low-E Coating Method	Window Type	Low-E Structure		−6 dB BW (GHz)	$\:{\boldsymbol{S}}_{21}$ (dB) at $\:{\boldsymbol{f}}_{0}$ (GHz)	$\:{{T}}_{{v}{i}{s}}({\%})$	$\:{{T}}_{{I}{R}}({\%})$	$\:{{\phi\:}}_{{m}{a}{x}}^{{T}{E}/{T}{M}}$ $(\:^\circ\:)$
^9^	Sputtering	Single-pane glass (BPF)	Multilayer coating (Oxide + Metal- NW Layers)		N/A	9 dB at 2.45 GHz	76	33.8	45$\:^\circ\:$
^14^	Sputtering	Double-pane glass (BSF)	Ag-based Low-E (Ag-nanowires)		36–37	5 dB at 36.5 GHz	39	18.6	N/A
^15^	Sputtering	Double-pane glass (BSF)	Ag-based Low-E (Ag-nanowires)		< 0.7–3.3	1.3 dB at 3 GHz	46	20	N/A
^28^	Etching	Double-pane glass (BPF)	Surface-mounted meta surface		4.25–5.12	17 dB at 4.9 GHz	< 61	N/A	60$\:^\circ\:$
This work	Screen printing	Single-pane glass (BPF)	ML		26.4–32.2	1.3 dB at 28 GHz	-	-	60$\:^\circ\:$
		Double-pane glass (BPF)	ML		26.4–29.4	0.9 dB at 28 GHz	-	-	
	Evaporation		PAP (Nano-nucleation)	TE coating	26.2–30.6	4.7 dB at 28 GHz	66	44	$\:60^\circ\:$
				LE coating	25.4–30.5	4 dB at 28 GHz	40	19	

**Fig. 20:**
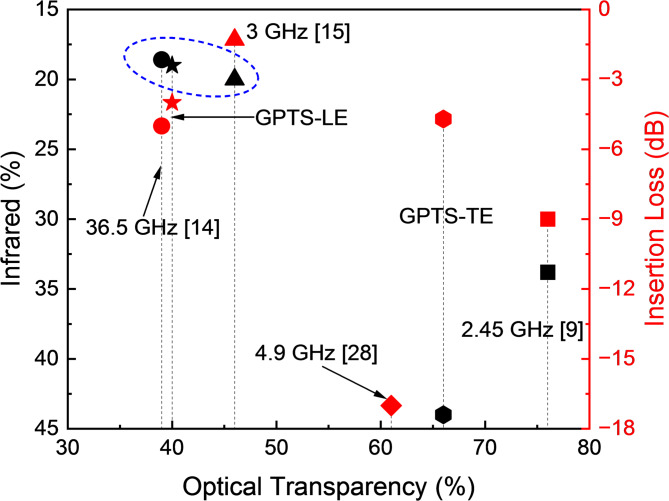
Comparison of $\:{T}_{\mathrm{vis}}$, $\:{T}_{\mathrm{IR}}$, and $\:{S}_{21}$ for the proposed double-pane GPTS-TE and GPTS-LE with previously reported in^9,14,15^, and^28^.

Table 5 summarizes and compares the key performance metrics of previous low-E glass studies with the proposed double-pane GPTS structures. The FSS in this work is designed as a bandpass filter targeting the n257 band, thereby enhancing frequency selectivity and effectively suppressing out-of-band interference^29^. In terms of physical properties, the TE coating based on the PAP structure exhibits superior optical performance compared with conventional DMD, Ag-based low-E, and multilayer oxide–metal coatings. As illustrated in Fig. 14a, under natural daylight the overall color difference between the coated and uncoated regions of the GPTS-TE window is minimal, indicating that the PAP-based coating has little visual impact in practical applications.

In terms of multispectral compatibility, previous works have been tailored to different wireless applications^9,15^: and^28^ focus on GSM mobile phones band or Wi-Fi systems, and^14^ considers a 36.5 GHz mmWave link. In contrast, this study is specifically optimized for the 28 GHz, aiming at 5G mmWave FWA and indoor–outdoor wireless communications. In Fig. 20, when the trade-off between $\:{S}_{21}$ and $\:{T}_{vis}$ is considered, the proposed GPTS-TE outperforms previously reported designs. Furthermore, when IR suppression is evaluated together with improved $\:{S}_{21}$, the GPTS-LE exhibits superior performance compared with^14^ in the mmWave band.

The selection of an optimal configuration depends on specific application requirements and trade-offs between electromagnetic and multispectral compatibility. For practical deployment, conventional low-E glazing products exhibit $\:{T}_{vis}$ values ranging from 17% to 70%, corresponding to solar transmittance values from 12% to 46%, depending on the desired level of energy efficiency^3^. For example, the EnerLogic VEP35 SR CDF film combines a low solar transmittance of 15–19% with a moderate $\:{T}_{vis}$ of 29–33%. Accordingly, this work provides detailed analyses of improving $\:{S}_{21}$ and $\:{T}_{vis}$, as well as optimizing $\:{T}_{IR}$ and $\:{T}_{UV}$, by varying the thickness and fill ratio of the coating layer in the proposed FSS structure.

## Conclusions

In this study, we developed three types of GPTS structures on double-pane glass using a metallic layer (ML), a transparent electrode (TE) film, and a low-emissivity (LE) film. The GPTS-ML exhibited near-ideal transmission performance (~ 1 dB at 28 GHz), approaching that of a perfect electric conductor (PEC). For GPTS-TE, removing 14.8% of the coating area (CRA) improved the transmission loss from − 19.5 dB to − 4.7 dB, with visible and infrared transmittance ($\:{T}_{\:vis}$ and $\:{T}_{\:IR}$) of 66% and 44%, respectively. Similarly, GPTS-LE reduced the transmission loss from 18.2 dB to 4 dB, while achieving excellent IR suppression ($\:{T}_{\:IR}$​ = 19%) and maintaining 40% $\:{T}_{\:vis}$. Overall, the proposed GPTS structures effectively balance electromagnetic compatibility and multispectral capabilities, making them promising candidates for enabling secure and efficient 5G mmWave wireless communications and FWA through low-E glass windows.

## Data Availability

The datasets used and/or analyzed during the current study are available from the corresponding author on reasonable request.
